# Prevalence and risk factors of post-traumatic stress disorder among elderly survivors six months after the 2008 Wenchuan earthquake in China

**DOI:** 10.1186/s12888-020-2474-z

**Published:** 2020-02-22

**Authors:** Lan Li, Jan D. Reinhardt, Craig Van Dyke, Heng Wang, Maoqiong Liu, Aiko Yamamoto, Qian Chen, Xiuying Hu

**Affiliations:** 1 Department of Nursing, West China Hospital, Sichuan University, Chengdu, China; 2 Department of Nursing, Southwest Medical University, Luzhou, China; 3 Institute for Disaster Management and Reconstruction of Sichuan University and Hong Kong Polytechnic University, Chengdu, China; 4 Swiss Paraplegic Research, Nottwil, Switzerland; 5 Department of Health Sciences and Medicine, University of Lucerne, Lucerne, Switzerland; 6 Department of Psychiatry, University of California San Francisco, San Francisco, CA USA; 7 Department of Anesthesiology, West China Hospital/West China School of Medicine, Sichuan University, Chengdu, China; 8 Department of Nursing, Dujiangyan Second People’s Hospital, Dujiangyan, China; 9 Research Institute for Nursing Care, Information Engineering National Institute of Technology, Nara College, Nara, Japan; 10 Center of Gerontology and Geriatrics, National Clinical Research Center of Geriatrics, West China Hospital, Sichuan University, No. 37 Guoxue Xiang, Chengdu, 610041 Sichuan Province China; 11 Innovation Center for Nursing Research, West China School of Medicine/West China Hospital, Sichuan University, No. 37 Guoxue Xiang, Chengdu, 610041 Sichuan Province China

**Keywords:** post-traumatic stress disorder, elderly, disaster, earthquake

## Abstract

**Background:**

Several studies indicate that older age is a risk factor for probable post-traumatic stress disorder (PTSD). However, evidence on the prevalence and risk factors for elderly disaster survivors is limited.

**Methods:**

Multi-stage stratified sampling was applied in this cross-sectional study. The Revised Version of the Impact of Event Scale (IES-R) was used to evaluate symptoms of PTSD. Chi-squared test was used for univariable analysis of prevalence of probable PTSD by possible determinants. Multivariable logistic regression analysis was utilized to further explore risk factors for PTSD.

**Results:**

427 elderly survivors age 60 or older participated. The prevalence of probable PTSD was 40.5, 36.3, and 21.5% according to a cut off score of the IES-R of 33, 35, and 2 points on average across items, respectively. In multivariable logistic regression, elderly survivors with a higher number of diagnosed chronic illnesses were more likely to be screened positive for PTSD and those with a greater number of family members living in the same home were less likely to be classified as having probable PTSD for all cut off scores. Elderly survivors with improved economic status and those with primary school or lower education were more often estimated to have probable PTSD with a cut off score of 35 and 2 points on average.

**Conclusions:**

Elderly survivors in a high impact area following a major earthquake are at high risk for developing PTSD. Those who live alone and report pre-existing chronic illness are particularly vulnerable.

## Background

Post-traumatic stress disorder (PTSD) is the most frequently reported mental disorder following disasters [1, 2] and was a major problem after the 2008 Wenchuan earthquake. A meta-analysis from 2016 by Dai and colleagues indicated that the pooled prevalence of PTSD from 46 studies of 16 earthquakes from eleven countries including 25 studies from the Wenchuan earthquake was 28.8% at 9 months or earlier and 19.5% after 9 months [3]. A recent systematic review of 58 studies of PTSD after the Wenchuan earthquake included 15 community studies on the prevalence of PTSD in the general population. Prevalence in the most severely impacted areas ranged from 86.2% in Mianzhu city at 1–2 months after the disaster to 8.0% in Yongang and Guangji township at 44 months after the earthquake. This reflects a pattern of declining prevalence of PTSD over time [4]. The same review also reported that being female, having lower education, being married, being middle-aged and older were demographic risk factors, while being trapped, suffering an injury, witnessing people dying, losing loved ones, and experiencing extreme fear during the disaster were additional risk factors. Feelings of guilt and low social support may also be associated with an increased risk of developing PTSD [3–5].

Older persons are particularly vulnerable to the adverse effects of disasters such as earthquakes. They may be at higher risk because of an accumulation of personal losses, decreased cognitive capacity, chronic illnesses, physical disability, social isolation, poor financial circumstances, limited access to resources, and communication difficulties in using modern technologies [6]. These chronic stressors may severely compromise psychological coping mechanisms, trigger memories of earlier traumatic experiences [7], and place the elderly at high risk for being emotionally overwhelmed following a destructive earthquake.

Previous research on PTSD after the Wenchuan earthquake focused on child and adolescent, community dwelling elderly survivors, and rescue workers. Huang and colleagues [8] surveyed 470 Wenchuan earthquake survivors residing in the community aged 60 years or older from three different areas, namely severely impacted (*n* = 101), moderately impacted (*n* = 153), and mildly impacted (*n* = 197) at 6 months after the disaster. They reported an overall PTSD prevalence of 32.9% with local prevalence estimates ranging from 56.3% in the severely impacted area to 25.6% in the moderately impacted area. They identified the following risk factors: belonging to an ethnic minority for the severely impacted area; being female and having experienced extreme fear during the earthquake for the moderately impacted area; and having a lower education level, having experienced fear during the earthquake, being religious, and having been injured or trapped for the mildly impacted area. Zhang and colleagues [9] conducted a community-based study including 284 survivors aged 60 years or older from severely impacted areas 1 year after the Wenchuan earthquake. They reported a prevalence of PTSD of 26.3% assessed with the PTSD Checklist. Loss of livelihood and initial level of fear were risk factors for PTSD among study participants. In addition, a study of 713 military veterans aged 60 years or older residing in the community of a general disaster affected area 2 years after Wenchuan earthquake found a PTSD prevalence of 3.09% as assessed with the PTSD Checklist. The incidence of angina, arrhythmia, hypertension, functional dyspepsia and sleep disorders was higher in elderly earthquake survivors with PTSD [10]. Another study reported a prevalence of 22.65% assessed with PTSD Scale for the Diagnostic and Statistical Manual of Mental Disorders in 287 Qiang citizens aged 60 years and older 3 years after the Wenchuan earthquake. They found the following risk factors for developing PTSD: being female, being widowed, having lower level of education, having lower monthly income, suffering an injury, being bereaved, and having lower level of social support [11].

Prior studies of PTSD prevalence and associated risk factors in the elderly following the Wenchuan earthquake have two limitations. Only one study was conducted about 6 months after the earthquake and sample sizes of elderly survivors from the severely impacted area were relatively small. Studying the prevalence and risk factors for developing PTSD in the elderly following natural disasters is important for several reasons. First, China has a rapidly aging population (National bureau of Statisics) with a 2017 estimate of 241 million people (17.3% of the population) age 60 years or older, and projections are that the elderly population will double by 2050. Second, PTSD causes marked suffering in individuals and families. It limits their functional capacity and compromises a community’s ability to recover from disasters [12, 13].

Our study aimed to: 1) estimate the prevalence of PTSD among elderly Wenchuan earthquake survivors in a severely impacted area at 6 months after the earthquake based on a representative sample, and 2) to determine possible risk factors within this population.

## Methods

### Design

This was a cross-sectional study conducted 6 months (November 2008) after the earthquake.

### Setting

An 8.0-magnitude earthquake occurred on May 12, 2008 in Wenchuan County of Sichuan Province, China. According to the Ministry of Civil Affairs, 69,227 people were confirmed dead, 374,643 injured, and an additional 17,923 were unaccounted for. Dujiangyan, a county-level prefecture and subdivision of Chengdu, was the fourth most severely impacted area in Sichuan Province. The intensity of the Wenchuan earthquake reached a peak of magnitude 9.0 on the Richter Scale and 3091 people were killed and another 141 were unaccounted for in this area [14]. On the basis of the disaster severity and impact, the State Council, divided the disaster area into most severely impacted areas (10 counties), severely impacted areas (41 counties), and general disaster areas (186 counties).

### Sample

A multi-stage stratified sampling method was applied. First, two counties were selected at random from Dujiangyan prefecture. Next, two villages were randomly chosen from each of the previously selected counties. Finally, two communities (neighborhoods) were randomly selected within the two villages and all elderly survivors were aproached in these two communities. In the following, elderly persons were approached at central places of the respective communities. Participants were encouraged to recommend other elderly persons living in their community to participate in the study who were then approached by the research team, i.e. employing a snowball method.

### Participants

Included were people aged 60 or older who lived in the selected communities, experienced the earthquake, and provided informed consent. Of 497 elderly survivors recruited for this study, 38 participants were excluded because they had difficulties understanding the questions. An additional 32 refused to participate. Thus, 427 participants completed the survey. The response rate was 85.9**%**.

### Data Collection

Two research assistants with master degrees in medical science were trained in the use of the IES-R and communication skills. The importance and purposes of this survey were explained to each participant, and they completed the scale based on the difficulty they experienced from the earthquake during the past 7 days. For those participants, who were unable to complete the questionnaire independently (e.g. limited literacy or physical illness) the research assistants read each question out loud, the participant answered verbally, and the assistants then registered their response on the questionnaire. After completion, data were checked for integrity.

### Variables

#### Outcome: Revised Version of the Impact of Event Scale (IES-R)

The IES-R is commonly applied to evaluate symptoms of PTSD [15]. The scale includes 22 questions and has three dimensions: intrusion, numbness/avoidance and hyperarousal. Participants are required to rank the severity of a particular event over the past 7 days on a scale ranging from 0 to 4 (none, few, moderate, high, extremely high). The total score ranges from 0 to 88. In China, a cut-off for IES-R has not been established for the general population or for a population of earthquake victims. The only study establishing a cut-off based on positive predictive value was conducted in a population of female offenders and the authors suggested a cut-off of 35 or 40 [16]. The comparability of the population examined in this research with our study population is, however, severely limited. No other studies using sensitivity, specificity, and/or positive predictive value of the Chinese IES-R to establish an optimal threshold exist to the best of our knowledge. While two studies on Wenchuan and Lushan earthquake victims used a cut-off of 35 based on the study of female offenders from Hunan women’s prison in China, other studies conducted in Chinese earthquake victims used a cut-off of 33 or of an average of 2 points across items based on validation studies of the English IES-R. The cut off scores used in different studies on Wenchuan earthquake survivors are provided in Table 1. In the present study, we provide results for cut offs of 33 and 35 total score as well as an average of 2 points across all scale items to assure optimal comparability with previous research.

**Table 1:** Different cut off scores of IES-R used for estimation of prevalence of PTSD in Wenchuan and Lushan earthquake survivors

Author (year)	Study population	Time point	Cut off score	Sample	Prevalence of PTSD
Qu et al. [17] (2012)	New mothers	8 months after 2008 Wenchuan earthquake	1.8	317	19.9%
Guo et al. [18] (2018)	Wenchuan Earthquake Survivors	8 years after 2008 Wenchuan earthquake	2.0	1369	11.8%
Guo et al. [19] (2017)	Wenchuan earthquake victims	8 years after 2008 Wenchuan earthquake	2.0	1369	11.8%
Guo et al. [20] (2015)	Wenchuan earthquake victims	6 months after event	2.0	1362	22.1%
Liu et al. [21] (2012)	Qiang Women	1 year after Wenchuan earthquake	2.0	270	37.0%(intrusion subscale), 26.3%(avoidance subscale), 32.6%(hyperarousal subscale) and 8.5%(all 3 subscales)
Qu et al. [22] (2012)	Pregnant women	1.5 years after Wenchuan earthquake	2.0	311	12.2%
Chan et al. [23] (2011)	Adult survivors	7 and 8 months after Wenchuan earthquake	2.0	243	55.6 and 26.4%
The cut off scores listed above describe average scores for each symptom/per symptom, whereas the ones below describe the overall summed score.					
Zhang et al. [24] (2011)	Adult survivors	2 months after Wenchuan earthquake	33	512	82.6%
Wang et al. [25] (2011)	Adult survivors	3 months after the 2008 Wenchuan earthquake	33	3622	31.4%
Wang et al. [26] (2010)	Health care workers	3 months after Wenchuan earthquake	33	343	19.0%
Xiuying Hu et al. [27] (2016)	Disaster-bereaved survivors	6 months after Wenchuan earthquake	33	226	38.9% at 6 months and 16.8% at 18 months
Ellen J. Schenk et al. [28] (2017)	Medical rescue workers	14 months after Wenchuan earthquake	33	337	17.0%
Chen et al. [29] (2015)	Elderly survivors	6 months after Lushan earthquake	35	1509	5.2%
Huang et al. [8] (2009)	Elderly survivors	6 months after Wenchuan earthquake	35	470	32.9%
Pan et al. [30] (2015)	Junior high school students	3 years after Wenchuan earthquake	40	373	29.6%

While an optimal cut off has not yet been established, the IES-R is one of two screening instruments for PTSD (the other is the PTSD checklist – civilian version) that have been translated and validated in Chinese. The Chinese version of the IES-R demonstrated good reliability and convergent validity based on its correlation with the General Health Questionnaire-20 [31] and can be used to assess the impact of traumatic life events in the Chinese population. Cronbach’s alpha reliability was reported as 0.890 in previous research [32]. In this survey, the value was 0.963.

### Probable risk factors of PTSD

Demographic variables assessed in this study included age, gender, marital status and educational level. Earthquake exposure related variables comprised: house damage, injuries to self and injury or death of family members (no injury vs. injury vs. death). Post-earthquake variables were change in economic status (worse than before, stayed the same, better than before); presence of chronic illnesses (Do you have one or more of the following health conditions? a) hypertension, b) diabetes, c) cerebral infarction, d) cancer, e) angina pectoris, f) asthma, or g) gout); and methods employed by the participants to evaluate their own health (How do you evaluate your own health? a) I regularly measure vital signs such as blood pressure, b) I regularly measure my weight, c) I regularly consult online resources and books about health issues, d) regularly visit Traditional Chinese Medicine doctors, and e) regular check ups at the hospital). The latter variable was supposed to indicate whether someone was more or less concerned about their overall health. Based on chronic illnesses reported, we created a sum index for the number of chronic illnesses for further analysis ranging from zero to seven. We also constructed a sum index for the number of methods employed by the study participants to evaluate their own health ranging from zero to five.

### Data Analysis

Stata (version 14.0) was used for all analyses. Descriptive statistics for the sample are provided. For further analyses, participants were divided into two groups based on their IES-R scores according to different cut off scores (i.e. total score ≥ 33, total score ≥ 35, and average score across items ≥2). Accordingly, the dependent variable was probable PTSD. Independent variables were age, gender, marital status, education level, house damage, number of people living in the same home, number of chronic illnesses reported (0, 1, 2 and more), number of methods employed by participants to evaluate their own health (0, 1, 2 and more). Univariable analyses were based on chi-square tests. Multivariable analysis was based on multiple logistic regression analysis. All variables were included in the multivariable logistic model since we were interested in exploring an overall predictive model, rather than precisely estimating the effects of particular variables. Two-sided testing was used for all tests, and the significance level was set at *P* <  0.05.

## Results

The mean age of the participants was 70.0 years (SD = 7.2) ranging from 60 to 91 years, 53.9**%** (*n* = 230) were male, 74**%**(*n* = 316) were married, and 66.7**%**(*n* = 285) had elementary school or a lower level of education. The prevalence of probable PTSD was 40.5, 36.3, and 21.5% with a cut off score of 33, 35, and 2 points on average across items, respectively (Table 2).

**Table 2:** Mean IES-R scores and prevalence of suspected PTSD by different cut off scores of IES-R (*n* = 427)

Variables	Probable PTSD according to IES-R n(%)		
	33 cut off score	35 cut off score	Cut off score average of 2 across items
Yes	173 (40.5)	155 (36.3)	92 (21.5)
No	254 (59.5)	272 (63.7)	335 (78.5)
IES-R	Mean ± standard deviation		
Intrusion	11.1 ± 5.9		
Avoidance	11.2 ± 6.5		
Hyperarousal symptoms	10.8 ± 5.8		
Total score	33.1 ± 17.4		

Table 3 presents the results for univariable analysis of probable PTSD by demographics and other potential risk factors. People aged 80 years and older had a higher prevalence of PTSD. Moreover, study participants whose economic status improved had a higher prevalence of PTSD than those whose economic status did not change or became worse. Those living alone and those reporting a higher number of chronic illnesses showed a higher prevalence of probable PTSD. Conversely, those not using any methods to evaluate their own health had a lower prevalence of probable PTSD than those employing one or more such means. Though not significant, the prevalence of PTSD in women was estimated about three to 4 % lower for cut off scores of 2 points per item and 33 points total score.

**Table 3:** Univariable analysis of probable PTSD by potential risk factors among elderly survivors six months after the Wenchuan earthquake (*n* = 427)

Variables	Subject, n(%)	Cut off score of 2 points on average			Cut off score of 33 total score			Cut off score of 35 total score		
		n(%)	X^2^	*P*	n(%)	X^2^	*P*	n(%)	X^2^	*P*
Age										
60–79	376 (88.1)	77 (20.5)	2.115	0.146	149 (39.6)	1.027	0.311	135 (35.9)	0.212	0.645
≥ 80	51 (11.9)	15 (29.4)			24 (47.1)			20 (39.2)		
Gender										
Male	230 (53.9)	46 (20.0)	0.703	0.402	89 (38.7)	0.683	0.408	83 (36.1)	0.010	0.921
Female	197 (46.1)	46 (23.4)			84 (42.6)			72 (36.5)		
Marital status										
Never married/divorced	14 (3.3)	2 (14.3)	1.104	0.576	3 (21.4)	2.252	0.324	3 (21.4)	1.413	0.493
Widowed	97 (22.7)	24 (24.7)			41 (42.3)			35 (36.1)		
Married	316 (74.0)	66 (20.9)			129 (40.8)			117 (37.0)		
Education level										
Elementary school or lower	285 (66.7)	69 (24.2)	3.797	0.051	124 (43.5)	3.340	0.068	114 (40.0)	5.059	0.024
Middle school	131 (30.7)	22 (16.8)			46 (35.1)			38 (29.0)		
Senior high school or higher	11 (2.6)	1 (9.1)			3 (27.3)			3 (27.3)		
Suffered own injury										
No injury	378 (88.5)	82 (21.7)	0.042	0.837	150 (39.7)	0.945	0.331	135 (35.7)	0.487	0.485
Injury	49 (11.5)	10 (20.4)			23 (46.9)			20 (40.8)		
Family members injured										
No injury	352 (82.4)	81 (23.0)	2.768	0.096	143 (40.6)	0.008	0.928	131 (37.2)	0.707	0.401
Injury	38 (8.9)	7 (18.4)			15 (39.5)			12 (31.6)		
Death	37 (8.7)	4 (10.8)			15 (40.5)			12 (32.4)		
House damage										
Slightly	84 (19.7)	17 (20.2)	0.106	0.745	28 (33.3)	2.233	0.135	28 (33.3)	0.397	0.529
Almost completely	343 (80.3)	75 (21.9)			145 (42.3)			127 (37.0)		
Change in economic status										
Improved	12 (2.8)	7 (58.3)	0.036	0.849	8 (66.7)	3.907	0.048	8 (66.7)	2.407	0.121
No change	228 (53.4)	43 (18.9)			77 (33.8)			69 (30.3)		
Declined	187 (43.8)	42 (22.5)			88 (47.1)			78 (41.7)		
Number of family members living in the same home										
0	151 (35.4)	49 (32.5)	16.38	< 0.001	76 (50.3)	10.210	0.001	69 (45.7)	10.271	0.001
1	113 (26.5)	21 (18.6)			44 (38.9)			40 (35.4)		
≥ 2	163 (38.2)	22 (13.5)			53 (32.5)			46 (28.2)		
Available measures to assess own health										
0	134 (31.4)	17 (12.7)	9.336	0.002	42 (31.3)	9.246	0.002	36 (26.9)	9.168	0.002
1	246 (57.6)	61 (24.8)			105 (42.7)			96 (39.0)		
≥ 2	47 (11.0)	14 (29.8)			26 (55.3)			23 (48.9)		
Chronic illnesses diagnosed										
0	224 (52.5)	29 (12.9)	19.939	< 0.001	64 (28.5)	29.490	< 0.001	56 (25.0)	25.338	< 0.001
1	133 (31.2)	40 (30.1)			67 (50.4)			63 (47.4)		
≥ 2	70 (16.4)	23 (32.9)			42 (60.0)			36 (51.4)		

The results of the multiple logistic regression analysis with cut off scores of 2 on average, and total scores of 33 and 35 are provided in Table 4. In all models the odds for being classified as having probable PTSD were lower with an increasing number of persons living in the household and were higher with the number of chronic illnesses. In addition, economic status was a significant predictor of probable PTSD in the model featuring a cut off of 2 points on average across IES-R items, indicating that people whose economic status did not change or improved showed higher odds of probable PTSD. The same trend was observed for the other cut offs. The odds for being classified as having probable PTSD also were lower with increasing educational achievement in the model employing a cut off score of 35. Age, gender, marital status, house damage, having suffered an injury, having family members who were injured or died as well as the number of methods participants employed to evaluate their own health status did not play a major role when it was adjusted for other covariates.

**Table 4:** Multivariable analysis of potential risk factors for probable PTSD for different IES-R cut off scores among elderly survivors six months after the Wenchuan earthquake(*n* = 427)

Variables	Cut off score of 2 points on average				Cut off score of 33 total score				Cut off score of 35 total score			
	OR	*P*	95% CI for OR		OR	*P*	95% CI for OR		OR	*P*	95% CI for OR	
			Lower	Upper			Lower	Upper			Lower	Upper
Age	1.5	0.271	0.7	3.2	1.5	0.245	0.8	2.9	1.2	0.678	0.6	2.3
Gender	1.1	0.809	0.6	1.8	1.1	0.598	0.7	1.8	0.9	0.816	0.6	1.5
Marital status	1.2	0.612	0.6	2.3	1.4	0.191	0.8	2.5	1.4	0.219	0.8	2.5
Education level (reference: elementary school or lower)												
Middle school	0.6	0.073	0.3	1.1	0.7	0.095	0.4	1.1	0.5	0.012	0.3	0.9
Senior high school or higher	0.3	0.324	0.04	2.9	0.5	0.314	0.1	2.0	0.5	0.361	0.1	2.1
Change in economic status (reference: improved)												
No change	0.2	0.022	0.1	0.8	0.3	0.082	0.1	1.2	0.3	0.074	0.1	1.1
Declined	0.2	0.029	0.1	0.9	0.5	0.261	0.1	1.8	0.4	0.198	0.1	1.6
House damage	1.2	0.572	0.6	2.3	1.5	0.134	0.9	2.6	1.2	0.523	0.7	2.1
Suffered own injury	0.9	0.848	0.4	2	1.3	0.460	0.7	2.4	1.2	0.551	0.6	2.3
Family members injured (reference: no injury)												
Injury	0.8	0.657	0.3	2	0.9	0.730	0.4	1.9	0.7	0.352	0.3	1.5
Death	0.4	0.116	0.1	1.2	0.9	0.741	0.4	1.8	0.7	0.383	0.3	1.5
Number of family members living in the same home	0.6	0.005	0.5	0.9	0.7	0.019	0.6	1.0	0.8	0.032	0.6	1.0
Chronic illnesses diagnosed	1.8	0.001	1.3	2.5	1.9	< 0.001	1.4	2.5	1.8	< 0.001	1.3	2.4
Available measures to assess own health	1.4	0.096	0.9	2.2	1.4	0.077	1	2	1.4	0.055	1.0	2.1
Constant	0.3	0.269	< 0.01	2.5	0.2	0.15	< 0.001	1.7	0.4	0.30	0.1	2.7

## Discussion

This research focused on the prevalence of PTSD and risk factors among elderly earthquake survivors from a severely impacted area 6 months after the Wenchuan earthquake. The prevalence of probable PTSD was high in this population. Participants with a higher number of self-reported chronic illnesses were more likely and those with a greater number of family members living in the same home were less likely to be screened positive for PTSD according to multivariable analysis, independent of the IES-R cut off score chosen. Elderly survivors with improved economic status and those with lower education level were more likely to be screened positive for PTSD in one of the models only. Differences in prevalence of probable PTSD by employing more methods to evaluate their own health were significant in univariable analysis only.

The prevalence of probable PTSD found in this study population when employing IES-R cut off scores of 33 or 35 was higher than the pooled prevalence reported in the meta-analysis on PTSD in earthquake survivors by Dai et al. [3] that indicated a pooled prevalence of probable PTSD of 28.8% at 9 months or earlier after earthquakes.

Since the prevalence of PTSD tends to decline with increasing time after earthquakes [3, 4], we focused our comparison on other studies that assessed PTSD prevalence 6 months after earthquakes. One previous study by Huang and associates reported the prevalence of probable PTSD in the elderly at 6 months after the Wenchuan earthquake, based on an IES-R cut off score of 35 [8]. The prevalence of probable PTSD reported by Huang et al. [8] for the severely impacted areas and the generally disaster affected area were about 10.0% lower than the prevalence reported in the present study. However, the prevalence for the most severely impacted area was about 20.0% higher than the prevalence found in the present study when using a cut off score of 35. It should be noted, however, that the sampling method employed in the study by Huang and colleagues was not specified. One possible explanation for the differences may be that the elderly were from Beichuan county, Anxian county, Shifang city and Dujiangyan city while all the participants in the present study were from Dujiangyan, that is closer to the capital Chengdu city, thereby allowing greater availability of medical and government support. Chen and colleagues [29] reported a prevalence of 5.2% probable PTSD with a cut off score of 35 among 1509 elderly survivors at 6 months after the Lushan earthquake, another severe earthquake that occurred in Sichuan province in 2013. This is markedly lower than our finding. A possible explanation is that the Wenchuan earthquake (magnitude 8.0) was more destructive than the Lushan earthquake (magnitude 7.0). Moreover, Lushan earthquake survivors may have benefited from experiences with psychological relief and medical aid gained during the Wenchuan earthquake. The prevalence of probable PTSD based on an IES-R cut off of 2 points on average across items found in the present study was similar to the one reported in a study on adult Wenchuan earthquake survivors by Guo and colleagues [20] which was conducted in a similar area. The 6 months prevalence of PTSD when based on an IES-R cut off score of 33 found in this study slightly exceeded the 38.9% prevalence reported at 6 months for a sample of 226 survivors whose family members had died in the earthquake [27]. However, the mean age of the participants in the former study was more than twenty years younger than our population.

Elderly survivors with more family members living in the same home had a lower prevalence of suspected PTSD in the present study. This result has not been explored in other studies but may reflect the beneficial effects of social support. Higher social support not only allows a greater opportunity to have contact with family members, friends, neighbors and other people, but also to get more resources from the household, community, society and the government. All these factors may help elderly survivors adapt to the changes in the environment caused by earthquakes. Elderly survivors with a high level of social support also tend to use more effective coping strategies than those with a lower level [33]. Elderly survivors with a higher number of family members living in the same home may not have experienced the earthquake alone. The feeling of fear and helplessness may thus be decreased. In line with the above considerations, intense fear was associated with less protective action to solve mental health problems [34] and increased traumatic stress symptoms following disaster in previous studies [35, 36]. Elderly survivors, who screen positive for suspected PTSD, and are living alone should be prioritized for receiving mental health service.

The result that female participants did not show a higher prevalence of probable PTSD was different from previous research. Chen and colleagues [11] found an association between being female and PTSD in 287 Qiang citizens aged 60 years and older 3 years after the Wenchuan earthquake. Being female was also a predictor of suspected PTSD in 360 participants, aged 18 years or older 3 years after the Wenchuan Earthquake [37]. Moreover, women were more affected by PTSD among 512 high school students 10 months after the L’Aquila 2009 earthquake in Italy [38].

Our finding that elderly earthquake survivors with a greater number of chronic illnesses were more likely to be screened positive for PTSD had not been reported previously. However, it has been demonstrated that the presence of chronic illnesses and the number of chronic illnesses can be associated with PTSD in non-disaster affected populations [39]. Another study from the Wenchuan earthquake also showed that the incidence of angina, arrhythmia, hypertension, functional dyspepsia and sleep disorders was higher in survivors with PTSD than in those without PTSD among elderly survivors 2 years after the disaster [10]. In the group of patients suffering from chronic disease, the hardship of a chronic disease itself, may contribute to a state of PTSD [40]. On the other hand, PTSD may contribute to the development of chronic physical conditions, e.g. by affecting lifestyles. Families, local health institutions and community leaders should be prepared for disasters to ensure that mental health treatment capacity is quickly resumed in disaster situations. Elderly disaster survivors with chronic medical conditions should be a priority target group for PTSD screening and treatment.

Surprisingly, Improvement in economic status was associated with an increase in suspected PTSD among elderly survivors in this research. This finding differs from prior studies. A meta-analysis of 37 studies with a total of 56,722 participants found that adult survivors with lower socio-economic status were more likely to suffer from PTSD after earthquakes [41]. Lower income [42] or economic hardship [43] was also related to a higher risk of psychological distress among survivors after the Great East Japan Earthquake. The current finding that the perception of improvement in economic status was associated with increased odds of probable PTSD is difficult to explain and the small number of survivors who indicated an increase in economic status leads us to interpret this result with caution. A study by Xu and colleagues showed that survivors with a higher monthly income were more likely to seek mental health services after the Wenchuan earthquake [44]. Accordingly, it is possible that those survivors were more aware of their symptoms. Moreover, it is also possible that those who received larger subsidies immediately after the earthquake were more severely affected by disaster-related stressors and had greater losses [45].

A trend for low educational level being associated with a higher prevalence of suspected PTSD among elderly survivors was observed in all models used in this study. This is consistent with previous research from the Wenchuan as well as 1999 Taiwan earthquake. A meta-analysis of predictors for PTSD in adult survivors after Wenchuan earthquake found that low educational level was a predictor of suspected PTSD in 30 of 37 studies that included this predictor [41]. Low education was associated with higher odds of PTSD among survivors from the general population both in the short- as well as long-term after the Wenchuan earthquake [46, 47]. Two other studies on elderly survivors of the Wenchuan earthquake also demonstrated that those having lower levels of education were more likely to develop suspected PTSD at 6 months [8] and 36 months after the disaster [11]. A possible explanation of this finding is that elderly survivors with higher educational level have more medical resources and information resources at their disposal and have a better economic status. Accordingly, they may be able to mobilize more resources and employ more successful strategies and thus be more resilient to the development of PTSD. As a result, elderly survivors with lower educational level should be particularly targeted after earthquakes in severely hit disaster areas. PTSD screening and mental health services from local hospitals should be given priority for them. In addition, family members should be made aware of how to recognize the symptoms of PTSD and should be included in the delivery of mental health services.

## Limitations

There are limitations to our study. First, the data was collected with a self-reported questionnaire that may have biased the results. The IES-R is a screening tool and cannot replace a diagnosis based on a systematic interview by a mental health professional. Second, no formal IES-R cut off score for PTSD has been established for a Chinese population. Accordingly, the prevalence of PTSD may have been over or underestimated. Third, baseline information on physical and mental health conditions in the population before the earthquake was not available so that excess morbidity in terms of PTSD due to the earthquake cannot be correctly determined. Fourth, since a control sample from a mildly or not affected area was not available, it is difficult to draw firm conclusions regarding of the effect of the earthquake on elevated prevalence of PTSD in elderly survivors. Fifth, although the sampling design was a multistage stratified design, we were unable to incorporate the strata into the analyses due to data restrictions. Sixth, as this study was conducted in a very severely impacted disaster area and the sample size was small, results may not be generalized to less severely impacted areas. Seventh, the small sample size may also result in low power to detect effects and imprecision in the estimation of confidence intervals.

## Conclusions

Our findings suggest that elderly survivors in high impact areas have a high prevalence of PTSD following an earthquake. This is particularly true for those living alone and suffering from chronic medical conditions. Both of these factors will become more prominent over time. The Chinese elderly population grows and takes up a larger portion of the general population and there are fewer young people around to live with them and care for them, in part due to the several decades long one child policy [48]. These two factors also interact in that chronic medical conditions not only require greater care but they also create functional disabilities. Fewer young people around means these people get worse care for their medical conditions but there are also fewer people to assist them in overcoming the barriers and obstacles that are present in a damaged environment, especially following a disaster [49]. Following an earthquake, this population warrants systematic screening for PTSD to identify symptomatic individuals for referral to mental health services.

## Data Availability

The data of this survey is available from the corresponding author (Xiuying Hu) upon reasonable request.

## Abbreviations

IES-R: Revised Version of the Impact of Event Scale
PTSD: Post-traumatic stress disorder
